# Primary pulmonary diffuse large B cell lymphoma presenting with features of organizing pneumonia: a case report

**DOI:** 10.1186/s13256-024-04973-7

**Published:** 2025-01-31

**Authors:** Sunjie Li, Yige Huang, Linlin Wang, Jintao Zhou

**Affiliations:** 1 Suzhou Medical College of Soochow University, Suzhou, 215000 People’s Republic of China; 2 Department of Respiratory and Critical Care Medicine, Taicang Hospital Affiliated With Soochow University, Taicang, 215400 People’s Republic of China

**Keywords:** Primary pulmonary lymphoma, Diffuse large B cell lymphoma, Organizing pneumonia

## Abstract

**Background:**

Primary pulmonary lymphoma is a rare subtype of non-Hodgkin lymphoma. Primary pulmonary diffuse large B cell lymphoma is an exceptionally rare form of primary pulmonary lymphoma. The clinical presentation of primary pulmonary diffuse large B cell lymphoma is often nonspecific, and imaging findings lack pathognomonic features, leading to frequent misdiagnosis and delayed treatment.

**Case presentation:**

An 81-year-old Chinese man presented with a 4-month history of pulmonary nodules and a 2-month history of cough and dyspnea. Initial empiric antibiotic therapy for suspected lung infection was ineffective. A chest computed tomography scan revealed multiple patchy and nodular opacities in both lungs. Bronchoscopy ruled out bacterial and fungal infections, leading to a diagnosis of organizing pneumonia. Treatment with systemic corticosteroids provided transient symptomatic improvement, followed by clinical deterioration. Upon admission to hospital, a percutaneous lung biopsy confirmed the diagnosis of primary pulmonary diffuse large B cell lymphoma. The patient was transferred to the hematology department and received rituximab combined with reduced dose cyclophosphamide, doxorubicin, vincristine, and prednisolone chemotherapy. Despite treatment, the patient’s clinical condition deteriorated, leading to eventual mortality owing to disease progression.

**Conclusion:**

Primary pulmonary diffuse large B cell lymphoma is a rare clinical condition with non-specific clinical manifestations, posing significant challenges for accurate and timely diagnosis. Early differentiation from other pulmonary conditions, such as organizing pneumonia, is critical to avoid delayed or inappropriate treatment, potentially improving patient outcomes. This case underscores the importance of a comprehensive diagnostic approach, including histopathological confirmation, in patients with pulmonary lesions that do not respond as expected to empiric therapies on the basis of preliminary diagnoses.

## Background

Primary pulmonary lymphoma (PPL) is a rare subtype of non-Hodgkin lymphoma originating from the lymphoid tissue of the bronchial mucosa or lung parenchyma. Primary pulmonary diffuse large B cell lymphoma (PPDLBCL) is an exceptionally rare form of PPL. The clinical presentation of PPDLBCL is often non-specific, and imaging findings lack pathognomonic features, leading to frequent misdiagnosis and delayed treatment. This case report describes a patient with PPDLBCL initially misdiagnosed as organizing pneumonia (OP), highlighting the diagnostic challenges and underscoring the importance of a comprehensive approach to evaluating pulmonary lesions with atypical features. By sharing this case, we aim to increase awareness among clinicians and improve the timely diagnosis and management of this rare disease.

## Case presentation

**Fig. 1:**
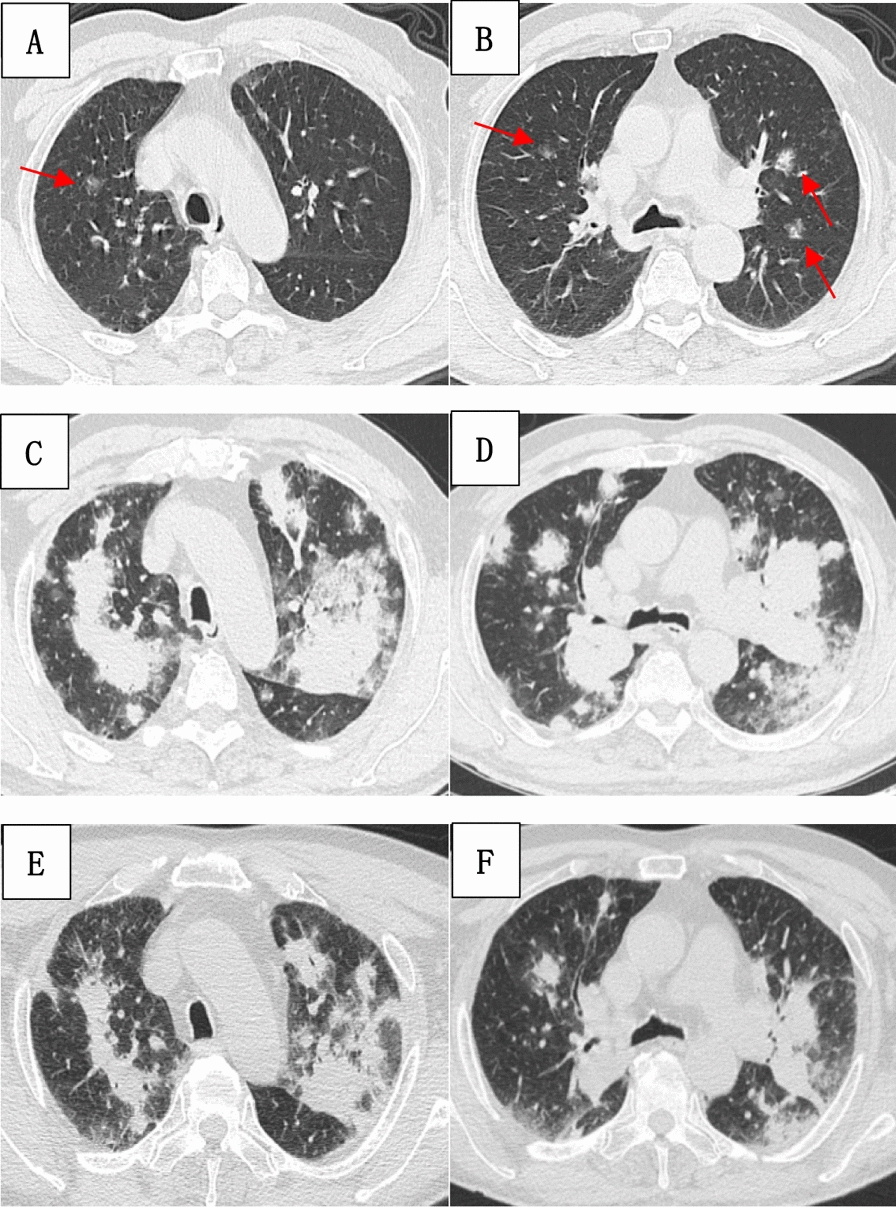
Chest computed tomography scan findings demonstrating the progression of lung lesions over time. (**A**, **B**) Initial presentation on 16 October 2023, with multiple small nodular shadows in both lungs (red arrows). (**C**, **D**) Progression to multiple consolidations and nodular high-density shadows on 21 December 2023. (**E**, **F**) Partial resolution of consolidations and nodular shadows on 20 January 2024

An 81-year-old Chinese man presented with incidentally discovered lung nodules over the prior 4 months and recent onset of cough and dyspnea persisting for 2 months. Initially asymptomatic, a routine chest computed tomography (CT) scan on 16 October 2023, revealed multiple small nodules in both lungs (Fig. 1A, B). He was prescribed a 4-day course of oral cefaclor and advised to return for follow-up evaluation, but he did not attend any subsequent appointments.

At 2 months prior to admission, the patient developed a new-onset cough, characterized by paroxysms and producing scant white sputum. He also experienced progressive exertional dyspnea. Notably, he denied fevers, chills, chest pain, or hemoptysis. A repeat chest CT scan performed on 21 December 2023, demonstrated progression of the lung lesions, with the appearance of multiple patchy and nodular high-density shadows, suggesting an infectious or inflammatory etiology (Fig. 1C, D).

Upon admission to the Department of Pulmonary Medicine of a local community hospital, the patient received empirical therapy with moxifloxacin, along with expectorants, antitussives, and bronchodilators. Arterial blood gas (ABG) analysis on room air revealed a primary respiratory alkalosis with hypoxemia, characterized by an elevated pH (pH 7.46), normocapnia (pCO_2_ 36.7 mmHg), and a reduced partial pressure of oxygen (pO_2_ 63.5 mmHg). Laboratory investigations showed a white blood cell (WBC) count of 7.6 × 10^9^/L with normal neutrophil count (neutrophils 68.7%), normal hemoglobin (123 g/L) and platelet count (2.25 × 10^11^/L), and an elevated C-reactive protein level (16.5 mg/L). Immunological tests, D-dimer, liver and kidney function tests, electrolytes, erythrocyte sedimentation rate (ESR), carcinoembryonic antigen (CEA), carbohydrate antigen 19–9 (CA 19–9), and alpha-fetoprotein (AFP) were all within normal limits. Sputum smear microscopy for acid-fast bacilli (AFB) was negative, and cultures for bacteria and fungi were also negative. Additionally, blood tests for T-SPOT.TB (interferon-gamma release assay) and antinuclear antibodies were nonreactive. Bronchoscopy revealed unremarkable airway anatomy without mucosal lesions. Brush cytology of the right lower lobe bronchus and bronchoalveolar lavage (BAL) were performed. Cytological examination of the bronchial brushings showed no evidence of malignancy, and metagenomic next-generation sequencing (mNGS) of the bronchoalveolar lavage fluid (BALF) detected no pathogens. Both the BALF β-D-glucan (G test) and galactomannan (GM test) were negative, excluding invasive fungal infection.

Given the patient’s clinical history, the absence of identified pathogens on extensive microbiological evaluation, and the radiographic findings, a presumptive diagnosis of secondary OP was made. Treatment was initiated with intravenous methylprednisolone at a dose of 40 mg daily. Within 3 days, the patient experienced significant improvement in both cough and exertional dyspnea. A follow-up chest CT on 20 January 2024, revealed partial resolution of the previously noted pulmonary opacities (Fig. 1E, F).The patient was discharged with a prescription for oral methylprednisolone (20 mg daily) and instructed to attend regular follow-up appointments at the outpatient clinic for monitoring and further management.

Approximately 4 weeks after discharge, the patient experienced a relapse of worsening cough and dyspnea. On 19 February 2024, he presented to our hospital for further evaluation. Chest CT revealed multiple patchy shadows and nodules in both lungs, suggestive of infection. Compared with the chest CT obtained at the outside hospital on 20 January 2024, there was radiographic evidence of disease progression. The patient was admitted to the respiratory and critical care unit with a preliminary diagnosis of pneumonia. His medical history was significant for pulmonary thromboembolism and left lower limb intramuscular vein thrombosis of unknown etiology for 1 year. He was receiving 20 mg of rivaroxaban once daily and denied any other chronic medical conditions.

**Fig. 2:**
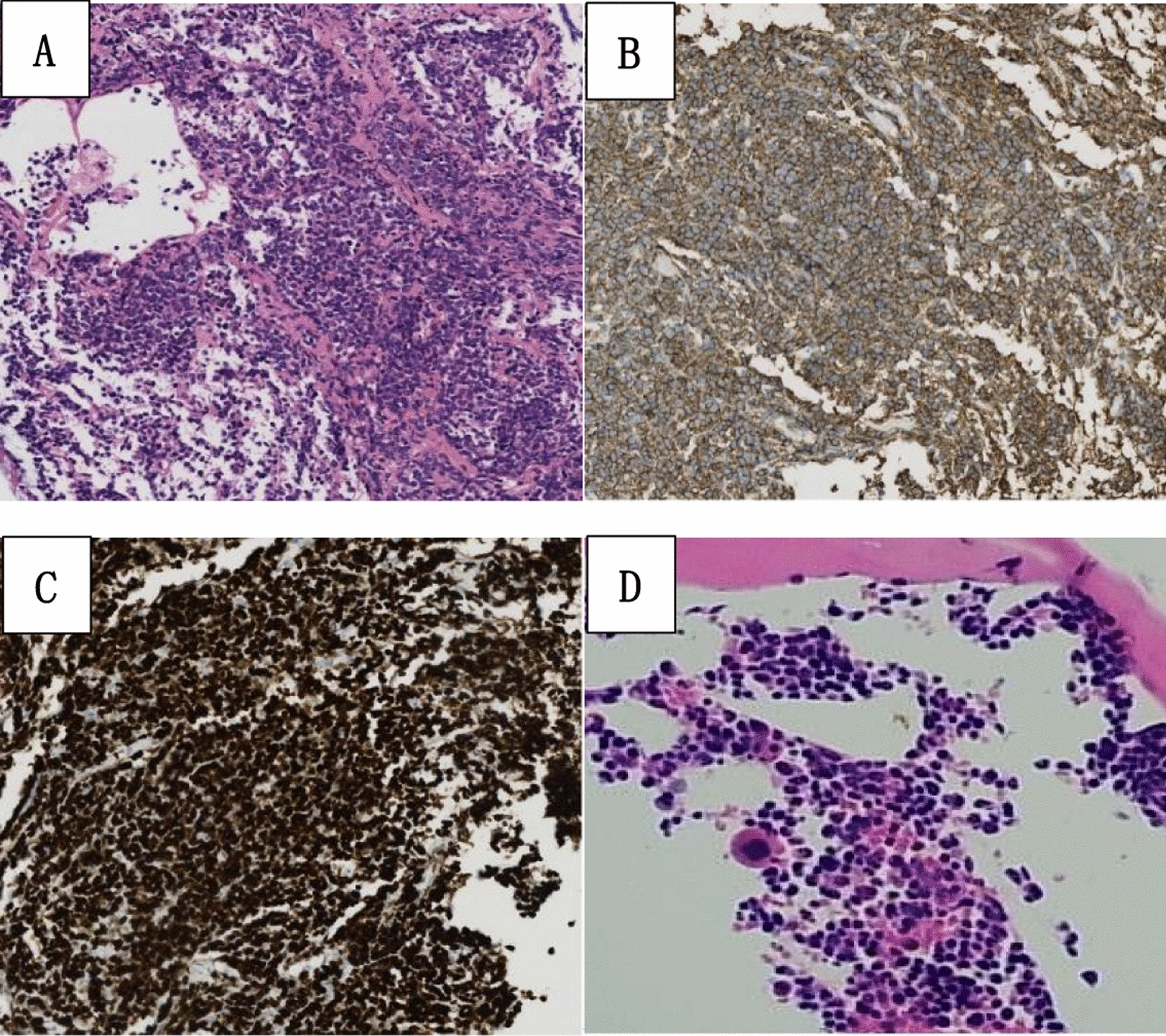
Hematoxylin and eosin staining of the lung biopsy demonstrate diffuse proliferation of large cells at lower power (10×, **A**). Immunohistochemistry staining (10×, **B**, **C**) of the lung biopsy shows positive for CD20 and Ki-67.Hematoxylin and eosin staining of the bone marrow demonstrate active hematopoiesis with an increased granulocyte ratio and decreased erythroid ratio (10X, **D**)

On admission, his vital signs were as follows: body temperature of 37 °C, heart rate of 63 beats/minute, respiratory rate of 21 breaths/minute, and blood pressure of 139/88 mmHg. The patient presented as alert and oriented, with no cyanosis of the lips or nailbeds. Respirations were mildly labored, with clear lung percussion notes on auscultation. Coarse breath sounds were noted bilaterally, but no adventitious sounds, such as crackles or wheezes, were appreciated. Cardiac examination revealed normal heart sounds, and no pathological murmurs on valve auscultation. Enhanced CT scans of the head, chest, abdomen, and pelvis upon admission demonstrated multiple patchy shadows and nodules in both lungs, suggestive of inflammatory lesions, along with hilar and mediastinal lymphadenopathy, coronary artery calcification, white matter attenuation, and cerebral atrophy. A comprehensive color Doppler ultrasound assessment of superficial lymph nodes revealed no abnormalities or enlargement. Laboratory findings included a white blood cell (WBC) count of 9.7 × 109/L with normal neutrophil count and an elevated lactate dehydrogenase (LDH) level of 378.9 U/L (Table 1). Following informed consent, a percutaneous lung biopsy was performed from the right lower lobe. Hematoxylin and eosin staining of the lung biopsy demonstrate diffuse proliferation of large cells at lower power (Fig. 2A). Immunohistochemistry and in situ hybridization analyses demonstrated tumor cells positive for cluster of differentiation 20 (CD20), CD19, paired box 5 (PAX5), CD10, B cell lymphoma 6 (Bcl6), and Bcl2 (approximately 80%). Additionally, the tumor cells showed variable expression of multiple myeloma oncogene 1 (about 30% positive cells), MYC (30–40%), and tumor protein 53 (P53) (20—30%). The tumor cells were negative for CD30, Epstein–Barr virus encoded RNA (EBER), cyclin D1, CD21, myeloid cell nuclear differentiation antigen (MNDA), CD3, and CD5, while exhibiting a high Ki-67 proliferation index (about 90%, Fig. 2B, C). Epithelial cells stained positive for cytokeratin AE1/AE3 (AE1/AE3). On the basis of these findings, the pathological diagnosis was diffuse large B cell lymphoma (DLBCL), specifically the germinal center B cell-like subtype. The patient met the diagnostic criteria for PPL and DLBCL, resulting in a final diagnosis of PPDLBCL. He was then transferred to the hematology department for further management.

**Table 1:** Laboratory results

Tests	Normal value	Results
WBC (10^9^/L)	4–10	9.7
Neutrophil count (10^9^/L)	1.8–6.3	6.1
Hemoglobin (g/L)	130–175	135
Platelet count (10^9^/L)	125–350	190
CRP (mg/l)	0–10	12.2
ALT (U/L)	0–40	22.1
AST (U/L)	0–40	15.7
Creatinine (umol/L)	40–120	88.1
LDH (U/L)	109–245	378.9
HBV DNA (IU/mL)	0–20	2
β2-microglobulin (mg/L)	1.3–3	3.58
aPTT (s)	23.3–32.5	25
D-dimer (mg/L)	0–0.55	0.35
HIV-antibodies		Negative
Epstein–Barr virus (EBV) IgM antibodies		Negative

*WBC* White blood cells,* CRP* C-reactive protein,* ALT* Associated lymphoid tissue,* LDH* Lactate dehydrogenase,* AST* Aspartate aminotransferase,* HBV DNA* Hepatitis bvirus deoxyribonucleic acid,* aPTT* Activated partial thromboplastin time,* HIV* Human immunodeficiency virus

**Fig. 3:**
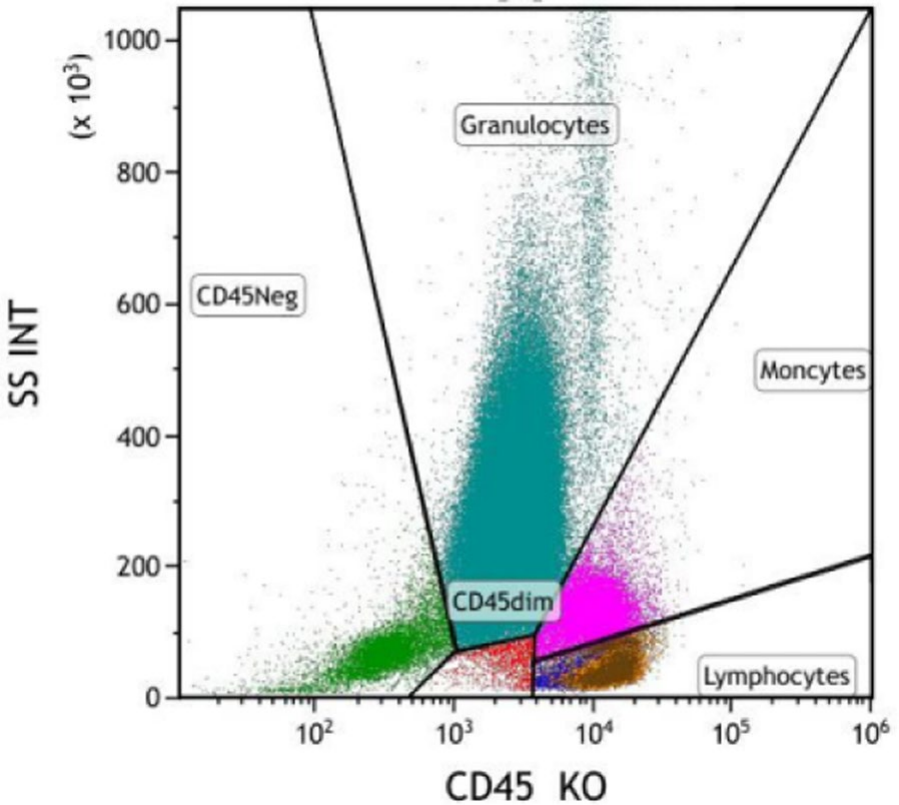
Flow cytometry of the bone marrow identified 0.32% CD5-/CD10- monoclonal lymphocytes

Bone marrow aspirate revealed active hematopoiesis with an increased granulocyte ratio and decreased erythroid ratio (Fig. 2D). Flow cytometry identified 0.32% CD5-/CD10- monoclonal lymphocytes (Fig. 3). Bone marrow biopsy demonstrated small clusters of CD20^+^ B lymphocytes (approximately 7%). Chromosomal karyotype analysis showed 45,X,-Y. The International Prognostic Index (IPI) score for lymphoma was 3, classifying the patient as high-intermediate risk. Following pretreatment assessment, the patient received rituximab and reduced dose CHOP (R-miniCHOP) chemotherapy (700 mg of rituximab on day 0, 0.7 mg of cyclophosphamide on day 1, 60 mg of doxorubicin on day 1, 2 mg of vincristine on day 1, and 10 mg of dexamethasone on days 1–5), along with supportive and symptomatic care. However, the patient’s condition deteriorated progressively, with follow-up chest CT showing further progression of lung lesions. At 2 weeks after initiating R-miniCHOP, the patient developed confusion and worsening dyspnea. Arterial blood gas analysis (while on 8 L/minute oxygen mask) revealed: pH of 7.35, pCO_2_ of 38 mmHg, and pO_2_ of 45.5 mmHg. Despite aggressive resuscitative measures, the patient ultimately succumbed to severe respiratory failure.

## Discussion

PPL is a relatively rare form of extranodal lymphoma, accounting for less than 1% of all primary malignant lung tumors and only 3.6% of extranodal lymphomas [1]. The most common histological subtype is mucosa-associated lymphoid tissue (MALT) lymphoma, representing 70–80% of cases, followed by DLBCL, accounting for 10–20% [2]. PPL is defined as clonal abnormal proliferation within the lung parenchyma or bronchi, with or without hilar lymph node involvement, and no evidence of extrathoracic lymphoma at diagnosis or within 3 months thereafter. While the traditional definition excludes extrathoracic disease, some researchers have broadened the criteria to allow for the presence of limited extrathoracic involvement during staging [3].

PPDLBCL typically affects middle-aged and elderly individuals, particularly those with compromised immune function, with no significant sex difference. Respiratory symptoms often include cough, dyspnea, and chest pain, with hemoptysis occurring less frequently. Systemic manifestations, such as fever, night sweats, and weight loss, are also common, but these are non-specific [4, 5]. Some patients may remain asymptomatic, with lung nodules or masses identified incidentally on routine imaging.

The radiographic presentation of PPL is diverse and lacks specificity, with solitary or multifocal nodules and consolidations being the most common findings. In PPDLBCL, approximately 50% of cases may present with solitary or multiple nodules [3], while ground-glass opacities and pleural effusion are less frequent [6]. In the present case, the patient was initially asymptomatic, with lung nodules detected incidentally on chest CT. As the disease progressed, he developed cough, sputum production, and dyspnea, with the lung nodules increasing in size and number and demonstrating coalescence. However, these clinical and radiographic features were non-specific, leading to an initial misdiagnosis.

The current diagnostic criteria for PPL include [5]: (1) histopathological evidence of lymphoma, (2) lesions confined to the thorax, and (3) absence of extrathoracic lymphoma within 3 months of diagnosis. In this case, lung tissue biopsy and immunohistochemistry confirmed DLBCL, consistent with the pathological findings of PPL. Imaging studies, including enhanced CT scans and ultrasound, revealed no evidence of extrathoracic involvement, thus fulfilling the aforementioned diagnostic criteria for PPL. Previous study suggests that for patients unable to undergo invasive lung biopsy, testing for mucosa-associated lymphoid tissue lymphoma translocation gene 1 (MALT1) rearrangement in BALF may offer a less invasive diagnostic approach [7].

Treatment of PPL should be individualized on the basis of patient age, performance status (PS) scale, International Prognostic Index (IPI) score, histological subtype, clinical presentation, and other relevant biological factors [8]. Prior to the 1990s, the CHOP regimen was the standard treatment for newly diagnosed DLBCL, but its efficacy was limited. The introduction of rituximab revolutionized treatment paradigms, with studies demonstrating that R-CHOP regimen significantly improved objective response rates (ORR) and overall survival (OS) compared with CHOP alone [9, 10]. Currently, R-CHOP remains the first-line therapy for patients with PPDLBCL [11]. In elderly patients over 80 years old, the R-miniCHOP may be considered [2].

In the present case, the initial misdiagnosis of OP led to treatment with methylprednisolone, a component of the R-miniCHOP regimen. The temporary clinical improvement and radiological response to corticosteroids further obscured the underlying lymphoma. While the patient ultimately received appropriate chemotherapy with R-miniCHOP, the delay in diagnosis and definitive treatment may have contributed to the unfavorable outcome. This case underscores the importance of early recognition and prompt initiation of appropriate therapy in PPL.

Several factors contributed to the misdiagnosis of PPDLBCL in this case. The patient’s initial presentation with cough and dyspnea was nonspecific, overlapping with common respiratory conditions, such as bronchitis and pneumonia. The radiographic findings of lung nodules and patchy shadows further mimicked those seen in pneumonia and OP, adding to the diagnostic challenge. Moreover, the initial response to glucocorticoid therapy, a treatment common to both PPDLBCL and OP, masked the underlying lymphoma. The rarity of PPDLBCL and limited clinician awareness of its varied presentations also played a role in the delayed diagnosis. Additionally, the patient’s non-compliance with recommended follow-up after initial treatment hindered early reassessment and potentially contributed to the missed diagnosis. This case highlights the importance of considering PPDLBCL in the differential diagnosis of pulmonary lesions and emphasizes the need for a comprehensive diagnostic approach, including histopathological confirmation, especially when the response to standard therapy is atypical or incomplete.

## Conclusion

Primary pulmonary DLBCL is a rare pulmonary malignancy with low incidence and non-specific clinical and imaging features, often leading to diagnostic challenges and potential misdiagnosis. When pulmonary lesions remain unexplained or fail to respond to therapy on the basis of the initial clinical impression, clinicians should broaden their differential diagnosis and consider less common conditions, such as PPDLBCL. In such cases, timely tissue acquisition through percutaneous or bronchoscopic biopsy is critical to establish a definitive diagnosis. This case underscores the importance of histopathological confirmation in the diagnosis of rare diseases and highlights the limitation of relying solely on clinical and radiographic findings. Furthermore, it emphasizes the need for heightened awareness of PPDLBCL among clinicians and reinforces the principle of judicious clinical practice, including the prudent use of glucocorticoids, to avoid obscuring the diagnosis of underlying malignancies.

## Data Availability

Not applicable.

## Abbreviations

PPL: Primary pulmonary lymphoma
PPDLBCL: Primary pulmonary diffuse large B cell lymphoma
OP: Organizing pneumonia
H&E: Hematoxylin and eosin
CT: Computed tomography
BALF: Bronchoalveolar lavage fluid
WBC: White blood cells
CRP: C-reactive protein
LDH: Lactate dehydrogenase
